# Immunometabolic effects of *β*-carotene and vitamin A in atherogenesis

**DOI:** 10.1097/IN9.0000000000000051

**Published:** 2024-11-28

**Authors:** Amparo Blanco, Jaume Amengual

**Affiliations:** 1 Department of Food Science and Human Nutrition, University of Illinois Urbana Champaign, Urbana, IL, USA

**Keywords:** apocarotenoid, forkhead box P3, retinoic acid receptor, retinoid, T regulatory cell

## Abstract

Carotenoids are a diverse group of lipids produced by photosynthetic organisms, and therefore, these compounds are major components of healthy diets. Carotenoids are among the most extensively studied micronutrients to date due to their antioxidant and provitamin A properties. *β*-carotene is one of the most abundant carotenoids in our diet, but more importantly, it is the main vitamin A precursor in humans. This review summarizes the key metabolic steps involved in vitamin A formation in mammals. It also highlights the recent advancements in the bioactive properties of *β*-carotene and vitamin A in relationship with atherosclerotic cardiovascular disease. We examine the dual effect retinoic acid, the transcriptionally active form of vitamin A, has on lipid metabolism and atherosclerosis development. Finally, we cover recent findings on the immunomodulatory role retinoic acid plays in macrophages and T cells in the context of atherosclerosis development and resolution.

## 1. Introduction to carotenoid metabolism

Carotenoids are a large group of tetraterpenoids typically produced by photosynthetic organisms. The unique absorption and light-emitting properties of carotenoids allow them to act as light scavengers to funnel photons toward the photosynthetic machinery. Some fungi also produce carotenoids. In these heterotrophic organisms, carotenoids also have a photoprotective role under stress conditions ^[1]^. Carotenoids function as “health” indicators for several species of birds, where they serve as indicators of physical fitness and reproductive potential ^[2]^. Many mammals also accumulate carotenoids, and these compounds serve as antioxidants and light scavengers. For example, humans selectively accumulate large amounts of lutein in the eye, where this carotenoid prevents blue-light damage and age-related macular degeneration ^[3–5]^.

Both photosynthetic and heterotrophic organisms cleave carotenoids to form signaling molecules named apocarotenoids. For example, plant-derived strigolactones modulate plant growth and communication with mycorrhizal fungi and parasitic weeds ^[6]^. Some fungi, such as *Fusarium spp*. produce provitamin A carotenoids and can synthesize retinal, the first vitamin A intermediate in mammals ^[7]^.

**Figure 1.**
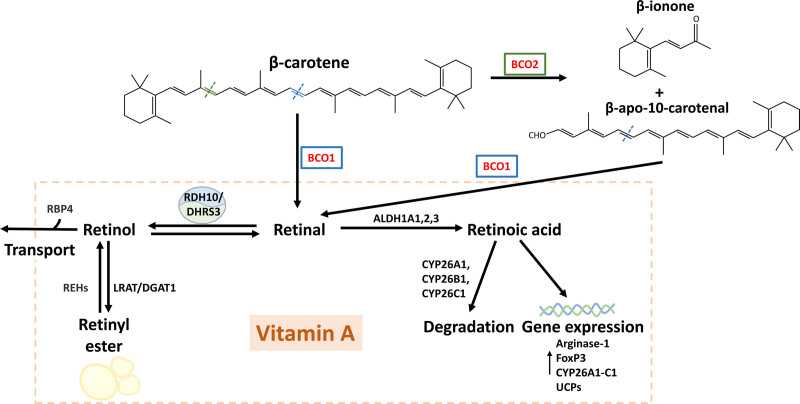
**Schematics of vitamin A formation in mammals.** ALDH1As, aldehyde dehydrogenase 1; BCO1, *β*-carotene oxygenase 1; BCO2, *β*-carotene oxygenase 2; CYP26s, cytochrome P450s; DGAT1, diacylglycerol *O*-acyltransferase; DHRS3, rehydrogenase reductase 3; FoxP3, forkhead box P3; LRAT, lecithin:retinol acyl transferase; RBP4, retinol-binding protein 4; RDH10, retinol dehydrogenase 10; REHs, retinyl ester hydrolases; UCPs, uncoupling proteins.

Unlike photosynthetic organisms and fungi, mammals express only two carotenoid-cleaving enzymes: the *β*,*β*’-carotene oxygenase 1 (BCO1) and BCO2 ^[8,9]^ (Figure 1). BCO1 is a cytosolic enzyme expressed primarily in enterocytes and hepatocytes ^[9,10]^, and the only enzyme responsible for vitamin A formation in animals ^[11]^. BCO1 is also expressed in other cell types such as the adipocyte, where it contributes to vitamin A production from carotenoid accumulated in fat stores ^[12,13]^. BCO2 localizes in the inner membrane of the mitochondria and has a broader tissue expression pattern than BCO1 ^[14]^. BCO2 prevents the accumulation of carotenoids in the mitochondria and their interference with the respiratory chain ^[15]^; however, whether BCO2 plays other roles in this organelle is still under investigation ^[15,16]^.

Carotenoids accumulate in human tissues and plasma in the micromolar range, which allows for their quantification using relatively simple techniques ^[17,18]^. The abundance of carotenoids in fruits and vegetables makes these compounds key components of a healthy diet. Among all carotenoids in nature, *β*, *β*’-carotene (*β*-carotene) is the most relevant for human health due to (1) its abundance in our diet and (2) the presence of two *β*-ionone groups in its structure that allow each *β*-carotene molecule to form two retinal molecules ^[19]^ (Figure 1). While we can obtain vitamin A directly from animal products, plant-derived carotenoids account for approximately 70% of vitamin A in people following an omnivore diet and are the only vitamin A source for strict vegetarians ^[20,21]^.

Association studies show clear correlations between increased plasma and tissue carotenoid levels and reduced occurrence of cardiometabolic diseases such as atherosclerosis, metabolic dysfunction-associated steatohepatitis, and obesity ^[22–24]^. This review focuses on the immunoregulatory effects of *β*-carotene and vitamin A in the context of atherosclerosis progression and resolution. We explore the mechanisms by which *β*-carotene, through its conversion to vitamin A and retinoic acid (RA), influences lipid metabolism in the liver and promotes an anti-inflammatory microenvironment in the atherosclerotic lesion.

## 2. Carotenoid and retinoid metabolism in mammalian organisms

### 2.1 Intestinal carotenoid absorption and vitamin A production

To sustain health, mammals must ingest either provitamin A carotenoids from plant sources or preformed vitamin A from animal sources. The uptake of preformed vitamin A as retinol and retinyl esters occurs by simple diffusion, which can lead to hypervitaminosis A ^[25]^. Conversely, carotenoids must be taken up by the scavenger receptor class B type 1 (SR-B1) expressed in enterocytes. The expression of SR-B1 and carotenoid uptake relies on a negative feedback loop regulated by RA levels. In the presence of vitamin A, the expression of the intestine-specific homeobox (ISX), an RA-sensitive gene, increases in enterocytes to bind the promoter region of SR-B1 and BCO1 to trans-repress their expression ^[26,27]^. Vitamin A deficiency results in a downregulation of ISX and an upregulation of SR-B1 and BCO1 to facilitate the uptake of carotenoids and the formation of vitamin A, respectively ^[28]^. Intact carotenoids and vitamin A in the form of retinyl esters are packed into chylomicrons and distributed to the lymphatic and circulatory systems (for a detailed overview of vitamin A and carotenoid metabolism in the intestine see ref ^[29]^.).

*β*-carotene is not the only provitamin A carotenoid in our diet. Asymmetric carotenoids such as *β*-cryptoxanthin and 9*-cis β*-carotene also play important roles in vitamin A homeostasis by undergoing sequential cleavage. First, BCO2 cleaves *β*-cryptoxanthin to generate all-trans *β*-apo-10-carotenal and 3-hydroxy ionone, or 9-*cis β*-carotene to form all-trans *β*-apo-10-carotenal and 9-*cis* *β*-ionone ^[11,30]^. Then, all-trans *β*-apo-10-carotenal, which contains a single *β*-ionone ring, serves as a BCO1 substrate to form a retinal molecule (Figure 1). This pathway is consistent with our recent studies on the fungal carotenoid neurosporaxanthin, a unique carotenoid with a *β*-ionone and a carboxylic group on each end of the molecule. We demonstrated that neurosporaxanthin is the preferred substrate for BCO1, showing for the first time it is a provitamin A molecule ^[31]^. In summary, BCO1 is the limiting step in vitamin A production, while BCO2 typically serves to degrade nonprovitamin A carotenoids, and in some instances, assists BCO1 in forming vitamin A from asymmetric compounds ^[11,32]^.

### 2.2 Overview of vitamin A metabolism

This section summarizes the key steps involved in vitamin A metabolism as highlighted in Figure 1. It is important to note that, although vitamin A contributes to cellular homeostasis in all cell types, the catalytic processes and proteins mentioned may not yet be fully understood in all cells.

#### 2.2.1 Retinal → Retinol

Retinal is the cleavage product of BCO1, and therefore, it is the first vitamin A intermediate (Figure 1). Retinal is also the visual chromophore in human photoreceptor cells, binding G-protein coupled receptors called opsins. In response to light, retinal isomerizes to initiate the signal transduction pathways responsible for vision ^[33]^. Like most aldehydes, retinal is highly reactive in the cellular environment, and therefore, it is quickly oxidized or reduced to RA or retinol, respectively. Therefore, retinal levels typically remain extremely low in cells other than ocular photoreceptors.

In most cell types, the conversion of retinal to retinol contributes to the regulation of RA levels. Studies by Kedishvili’s group described and characterized the presence of a bifunctional hetero oligomer formed between the dehydrogenase reductase 3 (DHRS3) and the retinol dehydrogenase 10 (RDH10) ^[34]^ (Figure 1). DHRS3 facilitates the reduction of retinal to retinol, while RDH10 acts as a key enzyme in the conversion of retinol to retinal. The formation of the DHRS3/RDH10 complex sustains the enzymatic activity of both enzymes to maintain retinoid homeostasis; however, the exact mechanisms underlying this cooperative effect are still under investigation ^[35,36]^.

#### 2.2.2 Retinol-binding protein 4 and vitamin A transport

The preferred transport form of vitamin A within the body is retinol bound to the retinol-binding protein 4 (RBP4). RBP4 is primarily produced by hepatocytes, but it is also expressed in other cell types such as adipocytes and macrophages ^[37]^. Because the liver is the primary vitamin A reservoir, hepatic RBP4 maintains vitamin A homeostasis by distributing vitamin A to extrahepatic tissues. Once RBP4 reaches a target cell, it interacts with the RA-sensitive membrane receptor stimulated by retinoic acid 6 (STRA6) ^[38]^. STRA6 expression is predominant in cells with high vitamin A requirements, such as the retinal pigmented epithelium in the eye ^[39]^, which explains why mutations in STRA6 typically result in microphthalmia and other congenital defects ^[40]^. A second RBP4 receptor named STRA6-like was identified in 2013 ^[41]^. STRA6-like displays an opposite distribution pattern to STRA6. For example, STRA6-like is expressed in the hepatocyte, where STRA6 is absent.

In 2005, Kahn’s group published a landmark paper describing the contribution of RBP4 to obesity and diabetes ^[42]^. This work was followed and validated by other studies showing positive associations between RBP4 levels in various cardiometabolic diseases ^[43–45]^. Adipocytes produce RBP4, and its levels typically increase with obesity, which leads to the hypothesis that adipose tissue and hepatic-derived RBP4 could play different roles in cardiometabolic diseases. Indeed, mice that over-express RBP4 in adipocytes also display hepatic steatosis ^[46]^. Data show that adipose-specific RBP4 secretion could act as a proinflammatory signal in macrophages ^[47]^, although these effects would be independent of STRA6 since macrophages do not express this receptor ^[48,49]^.

As a result of Kahn’s findings, the synthetic retinoid fenretinide gained popularity as a pharmacological strategy to reduce systemic RBP4 ^[50]^. Fenretinide mitigates cell proliferation, and it can be used to treat breast cancer. Fenretinide also binds RBP4 and favors its glomerular elimination by disrupting the ternary complex RBP4:retinol:transthyretin ^[51]^. Initial studies using fenretinide showed promising results in the treatment of cardiometabolic diseases; however, these effects could not be completely attributed to the reduction of RBP4 due to the stimulatory effects of fenretinide on retinoid signaling ^[52]^. Research by Amgen using a nonretinoid compound with high affinity for RBP4 failed to improve metabolic parameters such as glucose and insulin-tolerance tests in mice ^[53]^, curbing the enthusiasm for targeting systemic RBP4 to treat cardiometabolic diseases. For a recent overview of RBP4, see ^[54]^.

#### 2.2.3 Retinol → Retinyl ester

Besides the putative role of RBP4 as a signaling molecule, the main function of this protein is to serve as a retinol carrier. Once RBP4 interacts with STRA6, retinol must cross the plasma membrane to enter the cell ^[55]^. Following the law of mass action, the flux of retinol into the cell requires the presence of an intracellular retinol acceptor ^[56]^. In other words, STRA6 alone does not ensure that significant amounts of vitamin A will reach the cell unless retinol esterification occurs. This was evidenced in mice lacking the enzyme lecithin:retinol acyltransferase (LRAT), which exclusively catalyzes the esterification of retinol to retinyl esters ^[57]^. *Lrat*^*−/−*^ mice lack hepatic and ocular vitamin A, where LRAT is highly expressed. In the adipose tissue, where LRAT is not expressed, vitamin A stores are greater in *Lrat*^*−/−*^ mice than in their wild-type counterparts ^[58]^. Part of the vitamin A in the adipose tissue of *Lrat*^*−/−*^ mice appears esterified due to the activity of diacylglycerol acyltransferase-1 (DGAT1) ^[58,59]^, where it plays a key role in the formation of intracellular lipid droplets ^[60]^. In addition to its role in the esterification of diacylglycerols with an acyl-CoA to form triglycerides, DGAT1 also catalyzes the esterification of monoacylglycerols, waxes, and retinol. However, the retinyl ester activity of DGAT1 is lower in comparison to LRAT ^[61]^.

#### 2.2.4 Retinyl ester → Retinol

Retinyl esters are the main storage form of vitamin A in tissues, and several enzymes with hydrolase activity contribute to the mobilization of vitamin A. In the adipose tissue, adipose lipoprotein lipase ^[62]^ and hormone-sensitive lipase (HSL) ^[63,64]^ are the two main retinyl ester hydrolases. The hydrolysis of hepatic retinyl esters occurs primarily in hepatic stellate cells by the action of several enzymes such as lysosomal acid lipase ^[65]^, adipose triglyceride lipase ^[66]^, HSL ^[67]^, and other retinyl ester hydrolases ^[68,69]^. The pancreas also exhibits retinyl ester hydrolytic activity through the action of pancreatic triglyceride lipase ^[70]^.

Unlike LRAT, which is highly specific for retinol esterification, DGAT1 and retinyl ester hydrolases have broad substrate specificity and can be activated in response to fasting. Indeed, recent work by Schupp’s group highlights the role of adipose tissue HSL in maintaining vitamin A homeostasis during fasting conditions ^[71]^. They observed that fasting reduces the hydrolysis of hepatic retinyl esters to prevent the mobilization of vitamin A stores. In the absence of HSL, plasma, kidney, and ocular vitamin A are reduced during fasting, unveiling a previously unrecognized crosstalk between adipose tissue and the liver to maintain systemic vitamin A. This interaction emphasizes the role of adipose tissue in supporting systemic retinoid levels when hepatic mobilization is compromised, uncovering a more intricate mechanism of retinoid homeostasis ^[71]^.

#### 2.2.5 Retinal → RA

Three retinal dehydrogenases (ALDH1A1-A3) catalyze the irreversible oxidation of retinal to RA. ALDH1A1 is predominantly expressed in the liver, where it also catalyzes the oxidation of ethanol to acetaldehyde and acetate. Alcohol consumption leads to the depletion of hepatic vitamin A stores ^[72]^, possibly in part by the upregulation of vitamin A-catabolizing enzymes in response to alcohol exposure ^[73]^. ALDH1A1 is also expressed in adipocytes, where it sustains RA levels to influence energy balance, adipogenesis, and thermogenesis ^[74,75]^. ALDH1A2 is primarily expressed in the lungs, kidneys, and testes, while ALDH1A3 is primarily involved in ocular development and the development of certain tumors ^[76,77]^.

#### 2.2.6 RA levels and regulation of gene expression

All-trans RA (herein referred to as RA) binds and transactivates three retinoic acid receptor (RAR) isoforms (*α,* *β,* *γ*) to regulate the expression of approximately 700 genes. RA is the main form of vitamin A responsible for retinoid signaling during organogenesis and in adult organs. 9-*cis* RA binds both RARs and the three isoforms of retinoid X receptors (RXRs, *α,* *β,* *γ*). RXRs serve as obligate heterodimers of many nuclear hormone receptors, such as liver X receptors, vitamin D receptors, thyroid hormone receptors, and peroxisome proliferator-activated receptors, highlighting the interplay of vitamin A signaling in several aspects of gene regulation. Another ligand for RXRs is the 9-*cis* 13,14-dihydro RA. This compound was first identified by Moise and colleagues back in 2005 ^[78]^, but it was not until 2015 that it gained interest as the putative RXR endogenous ligand ^[79]^.

RA levels in tissues are in the picomolar range, making the detection of RA challenging using conventional instruments. Over the last decades, Napoli’s group perfected the quantification of RA using spectrometry-based techniques, which tremendously advanced the retinoid field ^[80]^. The detection of 9*-cis* RA was proven to be even more challenging than the detection of its isomer RA, and for many years, it was believed to be absent in biological samples ^[81]^. In 2010, Kane and colleagues demonstrated the presence of 9*-cis* RA in the pancreas and characterized its role in regulating glucose homeostasis ^[82,83]^. Recently, Napoli’s group optimized a novel methodology to detect 9*-cis* RA for the first time in tissues other than the pancreas, revealing that the occurrence of 9-*cis* RA is comparable to its all-*trans* isomer ^[84]^.

#### 2.2.7 RA degradation

Due to its profound effects at the gene expression level, intracellular levels of RA are tightly regulated primarily by three cytochrome P450 family members: CYP26A1, CYP26B1, and CYP26C1. These enzymes catalyze the oxidation of the *β*-ionone ring to form compounds such as 4-oxo RA, which facilitates its excretion in urine. The promoters of these genes contain several RA response elements to ensure a fast response to rising intracellular RA levels. The modulation of CYP26A–C1 varies in a cell type-dependent manner, and their mRNA levels are typically considered adequate, readily available surrogate markers to estimate RA levels in various experimental models ^[49,85–88]^.

## 3. Intact *β*-carotene as antioxidant

Humans accumulate large amounts of carotenoids in their plasma and tissues; however, the majority of experimental models in biomedical research fail to mimic this phenotype. This is also the case with wild-type mice, the most utilized animal model in biomedical research. Dietary studies using wild-type mice reveal that rodents are “great carotenoid cleavers,” failing to store carotenoids even after chronic feeding strategies with supra-physiological doses of carotenoids ^[89,90]^. Hence, wild-type mice are not an adequate model to study the biological effects of carotenoids in their intact form, nor the mechanisms regulating their distribution to tissues (reviewed in ref ^[91]^). Yet, studies in rodents indicate that *β*-carotene supplementation could mitigate lipid peroxidation ^[92–94]^, a driver of atherogenesis ^[95]^.

In 2007, von Lintig’s group developed *Bco1*^*−/−*^ mice, which accumulate intact *β*-carotene in plasma in their tissues ^[13]^. Since then, we have used *Bco1*^*−/−*^ and wild-type mice to dissect the relative contribution of intact *β*-carotene and its vitamin A derivatives in various cardiometabolic diseases ^[12,87,96,97]^. To date, we have reported protective effects of *β*-carotene supplementation in wild-type mice, but not in *Bco1*^*−/−*^ mice. These data suggest that intact *β*-carotene, regardless of its putative antioxidant properties, does not contribute to mitigation of cardiometabolic diseases in mice. Some clinical studies suggest that *β*-carotene supplementation does not mitigate lipoprotein oxidation ^[98–101]^, although others suggest that it can normalize markers of lipid peroxidation ^[102–105]^, Studies in ferrets, an animal model that resembles humans in their capacity to accumulate carotenoids in tissues ^[106]^, suggest that carotenoid supplementation strategies to mitigate oxidative damage is a viable approach to prevent and treat some cancers ^[107–109]^. Whether these approaches could also contribute to mitigation of cardiometabolic diseases remains unexplored.

With the premise that consumption of *β*-carotene could reduce the incidence of lung cancer in high-risk populations, the National Cancer Institute launched the Carotene and Retinol Efficacy Trial (CARET study) ^[110]^. A few years later, the study was halted early due to alarming findings that participants taking *β*-carotene had a significantly increased incidence of lung cancer—28%—compared to those who received a placebo ^[111]^. Similar conclusions were drawn from the Alpha-Tocopherol, Beta-Carotene Cancer Prevention Study (ATBC Study ^[112]^), raising serious concerns about the risks associated with *β*-carotene supplementation approaches that remain to this day. Careful evaluation of these outcomes suggested that the *β*-carotene dosage, which resulted in an approximately ten-time increase in plasma *β*-carotene levels, could be responsible for the negative health outcomes ^[113]^. A follow-up study of men enrolled in the ATBC Study revealed that increased circulating *β*-carotene baseline concentrations are associated with lower mortality, supporting the notion that increased levels of *β*-carotene from fruits and vegetables could promote longevity ^[114]^.

## 4. *β*-Carotene and vitamin A in atherosclerosis development and resolution

Atherosclerotic cardiovascular disease (ASCVD) is the leading cause of death worldwide ^[115]^. The underlying causes of ASCVD depend on an increase in proatherogenic lipoproteins in plasma that can lead to the dysregulation of the immune system ^[116]^. This section discusses the current evidence linking the effects of *β*-carotene and vitamin A on hepatic regulation of lipoprotein metabolism and atherosclerosis development. We will also cover novel findings connecting these nutrients in macrophage and T cell immunometabolism as key players in the progression and resolution of atherosclerosis.

### 4.1 Conversion of β-carotene to vitamin A in the regulation of the plasma lipid profile

Humans accumulate considerable amounts of *β*-carotene in their tissues and plasma, and genetic analyses have already described several single nucleotide polymorphisms (SNPs) for *BCO1*. Among the different variants, the SNP rs6564851 is among the best correlated with plasma *β*-carotene levels ^[117]^. This SNP localizes in the promoter region of BCO1, and studies by Lietz’s group showed that subjects harboring at least one copy of the rs6564851-T variant had increased BCO1 activity ^[118]^ (greater vitamin A formation). In 2020, we described the first association between BCO1 activity and plasma cholesterol in a cohort of young, healthy subjects. Our data showed a decrease of 10 mg/dL in total cholesterol and non-high-density lipoprotein cholesterol (non-HDL-C), cholesterol in subjects harboring at least one copy of the rs6564851-T variant, in comparison to individuals with two copies of the rs6564851-G variant ^[119]^.

These clinical data were supported by mouse studies comparing the effect of dietary *β*-carotene supplementation in wild-type and *Bco1*^*−/−*^ (normolipidemic) and low-density lipoprotein receptor (*Ldlr*^*−/−*^)-deficient mice and *Bco1*^*−/−*^*Ldlr*^*−/−*^ mice (hyperlipidemic) mouse models ^[96]^. In both studies, we observed that the effects of *β*-carotene in the regulation of plasma cholesterol were dependent on its conversion to vitamin A. Follow-up assays in cultured hepatocytes and mice exposed to RA suggested that the effects of *β*-carotene in the regulation of plasma lipids could depend on the production of RA and hepatic lipoprotein lipidation ^[96]^. These findings were in line with preclinical studies showing that *β*-carotene and RA favor fatty acid oxidation and inhibit lipid biosynthesis in hepatocytes ^[120–122]^. Altogether, such reduction could limit the lipid pool available for very low-density lipoprotein (LDL) assembly and secretion.

In 2010, Teslovich and colleagues ^[123]^ reported 95 loci associated with variations in blood lipids in a genome-wide association study with more than 100,000 individuals of European descent. Among those loci, they described an association between plasma triglycerides and the rs2068888 SNP, which is located in the promoter of the *CYP26A1* gene. Later, this SNP was also identified in other studies for not only triglycerides, but also LDL cholesterol (LDL-C), HDL-C, and total cholesterol ^[124,125]^. A mechanistic study by Napoli’s group identified the rs2068888 locus as a binding site for the transcription factor CCAAT/enhancer binding protein *β* (C/EBP*β*), which regulates basal CYP26A1 expression and RA levels ^[126]^. In our research, CYP26A1 is highly responsive to RA levels in metabolically relevant cells such as hepatocytes, myocytes, and adipocytes ^[12,87,88,122]^, which could explain the implication of CYP26A1 as a regulator of circulating triglyceride. Nevertheless, Napoli’s results agree with our data showing increasing RA levels can reduce the lipid content in newly secreted hepatic lipoproteins in both mice and cultured hepatocytes ^[96]^.

### 4.2 β-carotene and Vitamin A in atherosclerosis development

The first report describing the protective role of dietary *β*-carotene against atherosclerosis was published in 1995 by Shaish et al^[127]^ regarding hypercholesterolemic rabbits. Since then, clinical and preclinical studies have highlighted the relationship between *β*-carotene intake, plasma levels, and cardiovascular disease progression ^[89,97,114,128–130]^. Despite these advances, whether intact *β*-carotene, vitamin A, or both are responsible for delaying atherosclerosis remained unclear until we examined the effect of *β*-carotene supplementation in both *Ldlr*^*−/−*^ and *Bco1*^*−/−*^*Ldlr*^*−/−*^mice. By probing the effect of dietary *β*-carotene in the presence and absence of BCO1, we could dissect the relative contribution of *β*-carotene and its vitamin A derivatives and found that *β*-carotene decreases circulating cholesterol and delays atherosclerosis progression in *Ldlr*^*−/−*^ but not in *Bco1*^*−/−*^ *Ldlr*^*−/−*^ mice ^[96]^. This occurred despite *Bco1*^*−/−*^ *Ldlr*^*−/−*^ mice accumulating *β*-carotene in their plasma, liver, and other tissues, including the atherosclerotic lesion ^[96]^, as it occurs in humans ^[131]^.

**Figure 2.**
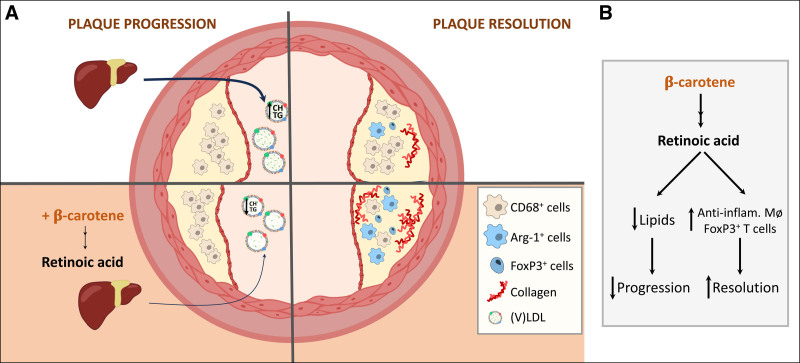
**Effect of *β*-carotene supplementation in the development and resolution of atherosclerosis.** (A) Changes in size and composition reported in the atherosclerotic lesion during atherosclerosis progression (left panels), resolution (right panels), in the absence (top panels) and presence (bottom panels) of dietary *β*-carotene. (B) Proposed mechanisms by which *β*-carotene modulates atherosclerosis.

Shaish’s group also reported that RA derived from 9-*cis* *β*-carotene inhibits foam cell formation in the macrophage ^[132]^. A follow-up study confirmed these findings and expanded them by using a combination of 9-*cis* *β*-carotene with the CYP26A-C1 inhibitor liarozole, reporting that the effects of 9-*cis* *β*-carotene in cultured macrophages. An important question that remains unanswered is: How do macrophages obtain, store, and utilize vitamin A? We have shown in various experimental models that macrophages express marginal levels of *BCO1* and lack both STRA6 and STRA6-like were due to increased RA levels ^[133]^. Whether 9-*cis* *β*-carotene cleavage results in the production of RA, 9-*cis* RA, or both remains unclear and will require further study. However, the effects of 9-*cis* *β*-carotene on macrophages could only be explained by the activity of BCO1. To test the contribution of BCO1 in macrophages in the progression of atherosclerosis, and whether these cells could rely on circulating *β*-carotene to form vitamin A in the atherosclerotic lesion, we performed a bone marrow transplant experiment. We transplanted bone marrow progenitor cells isolated from wild-type or *Bco1*^*−/−*^ mice into lethally irradiated *Bco1*^*−/−*^*Ldlr*^*−/−*^ recipient mice. After recovery, the recipient mice remained on a Western diet supplemented with *β*-carotene. Laser-capture microdissection to collect and purify mRNA from CD68^+^ cells in the atherosclerotic lesion, together with morphometric analyses, revealed that BCO1 expression was marginal in plaque CD68^+^ cells and that its deficiency in the myeloid lineage had only a minimal effect on atherogenesis ^[96]^ (Figure 2). Overall, our findings align with those reported by other groups in which *β*-carotene delays atherosclerosis progression by ameliorating the plasma lipid profile ^[89,96,127,128,134]^. To gain more information, *β*-carotene supplementation and the risk of cardiovascular disease have been recently reviewed in this meta-analysis ^[135]^.

### 4.3 Immunomodulatory effects of retinoic acid in macrophages

#### 4.3.1 Vitamin A homeostasis in the macrophage

An important question that remains unanswered is: How do macrophages obtain, store, and utilize vitamin A? We have shown in various experimental models that macrophages express marginal levels of BCO1 and lack both STRA6 and STRA6-like transporters ^[31,96]^. However, macrophages store both provitamin A carotenoids and retinyl esters ^[61,136]^, and murine bone marrow-derived macrophages (BMDMs) differentiated with regular growth media (Dulbecco's Modified Eagle Medium [DMEM] + 10% fetal bovine serum) store retinoids and release RA ^[49]^. Taking advantage of the phagocytic activity of macrophages, we established a methodology to load macrophages with vitamin A using lipoproteins isolated from animals fed high doses of retinyl ester ^[137]^. Our assays showed that macrophages fail to take up free retinol from the media, suggesting that, as it occurs with other cell types, vitamin A uptake in macrophages depends on the principle of mass action to prevent the oxidation or degradation of intracellular retinoids. When using lipoproteins enriched with vitamin A, this protocol enables us to track the fate of vitamin A in the macrophage, including vitamin A secretion, as is routinely done for cholesterol efflux assays ^[138]^. Once the lipoproteins are taken up, retinyl esters contained primarily in chylomicrons are hydrolyzed in the lysosome and re-esterified by DGAT1 ^[61]^. Nonetheless, the relatively low affinity of DGAT1 for retinol in comparison to LRAT ^[56]^, which is not expressed in the macrophage ^[31]^, explains the limited capacity of macrophages to store vitamin A.

The fate of vitamin A stored in macrophages is a current research topic in our lab. Our unpublished data show that vitamin A-loaded macrophages excrete the majority of vitamin A quickly, suggesting that these cells are not capable of storing vitamin A for a long period, unlike the stellate cells in the liver ^[139]^. The high hydrolytic capacity, combined with the limited DGAT1 activity to esterify retinol, may explain the limited capacity of the macrophage to store vitamin A, suggesting that the fast vitamin A turnover in these cells would require a constant influx of vitamin A into the cell to ensure vitamin A homeostasis and cellular responses.

In 2010, Broch et al ^[37]^ described that macrophages express and release RBP4, suggesting that these cells could supply neighboring cells with vitamin A. We recently showed that the anti-inflammatory cytokine IL4 upregulates RBP4 expression in murine macrophages, although our studies using radiolabeled retinol demonstrate that IL4 exposure does not result in a net increase in RBP4-retinol secretion. IL4 also promoted the reduction of retinyl ester stores in the macrophage, which was accompanied by the upregulation of the lysosomal acid lipase, a retinyl ester hydrolase ^[65]^. Importantly, we reported for the first time that IL4-exposed macrophages produce and release RA ^[49]^. These effects were mediated by the upregulation of the RA-producing enzyme ALDH1A2, as previously suggested by other groups ^[140–143]^.

#### 4.3.2 Role of RA in macrophage polarization

Macrophages play a critical role during atherogenesis. In response to environmental signals, tissue (and plaque) resident macrophages can polarize toward a variety of proinflammatory (classical) and anti-inflammatory (alternatively activated) phenotypes that will dictate their role in the atherosclerotic lesion by favoring either inflammation or plaque stabilization, respectively ^[144]^.

We, and others, have shown that RA treatment can modulate the polarization status of cultured macrophages by regulating gene expression. For example, RA is commonly used to switch macrophages from a proinflammatory to an anti-inflammatory phenotype, contributing to the resolution of inflammation and tissue repair ^[145–149]^. While cell culture studies have limitations, a clinical study suggested that *CYP26B1* activity, a major RA catabolic gene, is associated with atherogenesis in humans. The authors observed that the rs2241057-G SNP variant, which presumably results in a greater CYP26B1 activity (lower RA levels), is associated with larger atherosclerotic lesions in a small cohort ^[150]^. The exact mechanism responsible for increased atherogenesis in response to higher CYP26B1 remains unclear, but it indicates that higher RA levels in the lesion could promote an anti-inflammatory phenotype in plaque macrophages. While CYP26A1 is the major catabolic enzyme in most cell types, our results show that only CYP26B1 responds to RA in BMDMs ^[49]^. It is also possible that increased RA levels in plaque macrophages limit the development of cholesterol-laden foam cells by upregulating the ATP-binding cassette transporter 1 (ABCA1), an RA-sensitive gene ^[151]^, to promote cholesterol efflux to nascent HDL ^[151–154]^.

### 4.4 Immunomodulatory effects of retinoic acid in T cells

T cells are key mediators of the development and resolution of atherosclerosis. While proinflammatory T helper (T_H_)1 and T_H_17 cells contribute to plaque growth and instability, regulatory T cells (Tregs) counteract inflammation and promote plaque stability by suppressing the activity of proinflammatory T cells and macrophages ^[155]^. Tregs were first localized in atherosclerotic plaque by Ait-Oufella’s group ^[156]^, who showed them to have atheroprotective properties. Later, other groups explored the role of Tregs as anti-inflammatory agents present in regressing plaques. Tregs actively contribute to the resolution of inflammation by suppressing proinflammatory responses and promoting anti-inflammatory macrophage phenotypes, facilitating tissue repair and plaque stabilization ^[157,158]^.

#### 4.4.1 Role of RA in Treg differentiation

In 2003, three independent groups demonstrated that the differentiation of naïve T cells into Tregs requires the expression of the forkhead box P3 (FoxP3) ^[159–161]^. FoxP3 is necessary and sufficient to differentiate T cells into Tregs and serves as a lineage specification factor regulator of the expression of immunosuppressive genes ^[159,162]^. Subsequent research on oral tolerance demonstrated that RA upregulates FoxP3 expression. Mucosal dendritic cells in gut-associated lymphoid tissue synthesize RA to promote the differentiation of naïve T cells to Tregs, which helps to maintain gut-barrier integrity and immune response against pathogenes ^[163–167]^.

#### 4.4.2 Vitamin A homeostasis in T cells

The mobilization of free retinol from vitamin A stores is a major step in the regulation of RA levels, and therefore, vitamin A signaling. In T cells, this possibility was first explored by Graham et al ^[168]^ where they showed that the depletion of DGAT1 in these cells promotes Treg expansion by blocking retinol esterification, which resulted in greater RA production. Conversely, Howie et al ^[169]^ argued that DGAT1’s role in regulating lipid droplets is critical for maintaining Foxp3 expression and Treg stability. The authors propose that Tregs require DGAT1 to protect the cell against lipotoxicity and to limit protein kinase C activity, which can inhibit FoxP3 transcription. These discrepancies could be attributed to experimental approaches, and future experiments are required to establish the contribution of DGAT1 and vitamin A stores to naïve T cells and their role in Treg skewing.

The mechanisms responsible for vitamin A uptake in T cells remain elusive but seem independent of STRA6 ^[170]^. In 2021, Bang et al ^[171]^ showed that the serum amyloid A produced in the enterocyte delivers retinol to myeloid cells by interacting with LDLR-related protein (LRP1). It is plausible that this mechanism facilitates the uptake of vitamin A by T cells, as these cells also express LRP1 ^[171]^. It remains unclear what factors regulate vitamin A homeostasis in naïve T cells, and whether intracellular or extracellular RA is responsible for Treg skewing. The current evidence suggests that T cells may depend on exogenous RA, including anti-inflammatory macrophages, to differentiate into Tregs ^[97,141]^. Under these conditions, whether macrophage-derived RA enters naïve T cells remains unclear since serum amyloid A and RBP4 do not transport RA ^[56,171,172]^.

### 4.5 Dietary β-carotene in atherosclerosis resolution

While numerous studies have explored the effects of *β*-carotene and RA on atherosclerosis progression, our group reported for the first time the effect of dietary *β*-carotene on atherosclerosis resolution. To do so, we used two independent murine models in which we developed atherosclerotic lesions by inducing hypercholesterolemia and later normalizing plasma lipids either by switching the mice to a standard diet or by recovering LDLR expression in the presence or absence of dietary *β*-carotene. In both models, dietary *β*-carotene favored atherosclerosis resolution, reported by a decrease in CD68^+^ cells and an increase in collagen in the lesion. Importantly, the effect of *β*-carotene was abrogated in *Bco1*^*−/−*^ mice, further implying vitamin A as the key regulator of this process ^[97]^.

Unlike in our progression study ^[96]^, dietary *β*-carotene did not ameliorate plasma lipid parameters ^[97]^. Therefore, we explored whether dietary *β*-carotene could enhance Treg production and whether these cells were responsible for the effects of *β*-carotene during resolution. We accomplished this by injecting an antibody targeting CD25^+^ cells, a common marker of Tregs ^[157,173]^. While this approach failed to completely deplete Tregs by sparing CD25^−^ FOXP3^+^ Tregs, this strategy was sufficient to mitigate the effects of dietary *β*-carotene on atherosclerosis resolution ^[97]^. To our knowledge, this study remains the only experimental strategy in which a single nutrient promoted Treg expansion in the atherosclerotic lesion. We also observed an increased number of anti-inflammatory macrophages in the lesion accompanied by increased CYP26B1 levels in those mice fed *β*-carotene, regardless of our anti-CD25 treatments. Overall, our data show that dietary *β*-carotene promotes plaque stabilization in a Treg-dependent manner and that RA signaling in the plaque is not sufficient to promote atherosclerosis resolution in the absence of Tregs (Figure 2).

## 5. Future approaches and cautionary notes

**Figure 3.**
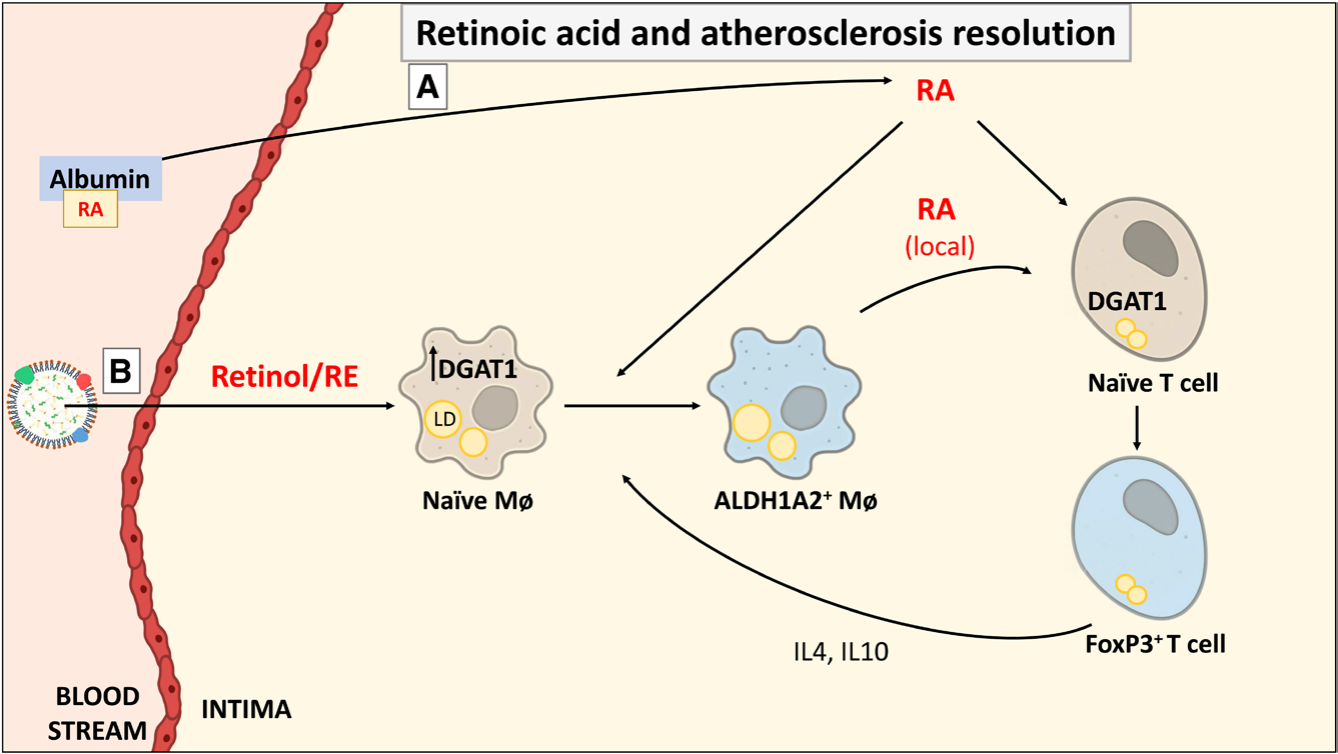
**Proposed immunomodulatory effects of RA during atherosclerosis resolution.** We propose that naïve macrophages receive vitamin A by (A) direct diffusion of retinoic acid (RA) into the lesion, and/or (B) engulfing lipoproteins containing vitamin A. Blue cells represent anti-inflammatory cells. ALDH1A2, retinaldehyde hydrogenase 1 family member 2; DGAT1, diacylglycerol *O*-acyltransferase; FoxP3, forkhead box P3; IL4, interleukin 4; IL10, interleukin 10; LD, lipid droplet; Mø, macrophage; RA, retinoic acid; RE, retinyl ester; Treg, T regulatory cell.

Our efforts are currently focused on establishing the role and the source of vitamin A in the crosstalk between macrophages and T cells in the atherosclerotic lesion. The status of vitamin A in the lesion, establishing the cell(s) responsible for RA secretion in the plaque, and the effect of sustained or intermittent vitamin A deficiency, among other factors, will be crucial to understanding the influence of *β*-carotene and vitamin A in the progression and resolution of ASCVD (Figure 3). Although the role of retinoids in hematopoiesis and differentiations was established decades ago, recent studies in high-impact journals highlight the relevance of studying RA in biomedical research. For example, Cabezas-Wallscheid’s group showed that RA regulates stem cell stem differentiation and survival during stress ^[174,175]^. Similarly, Fuchs’ team described that RA signaling dictates the lineage plasticity of hair follicle stem cells, balancing between maintaining stem cell identity and enabling differentiation during wound healing and the phagocytic capacity of cells in the hair follicle ^[176,177]^. In the context of tumor development and microenvironment, however, RA promotes differentiation toward an anti-inflammatory phenotype, which is often associated with tumor-supportive functions and growth ^[178]^. Further studies will be required to underpin the role of *β*-carotene and BCO1 as the limiting step in vitamin A formation, a nutrient-like hormone with major downstream effects in ASCVD and other diseases.

Experimental approaches using bioactive molecules require careful experimental designs to avoid confounding factors. For example, some studies infer the effects of *β*-carotene on cardiometabolic health parameters by using a diet of carrots, which are rich in this carotenoid. However, a recent study revealed the presence of a previously uncharacterized bioactive compound in carrots that could partially mediate the benefits previously attributed to *β*-carotene ^[179]^. Another important factor to consider is the dosage of these bioactives. Because mice do not accumulate carotenoids efficiently due to the expression of BCO1 and BCO2 and are resistant to retinoid toxicity, several groups utilize pharmacological and potentially toxic dosages of carotenoids and retinoids to study cardiometabolic diseases (reviewed in ref ^[91]^.).

## Conflict of interest

The authors declare no conflict of interest.

## Funding

This work was funded by the National Institutes of Health (R01HL147252) and the United States Department of Agriculture (W5002) to J.A.

## Acknowledgments

We thank Kate Epstein for her help editing the manuscript.
